# 4-Aminobenzoic Acid Derivatives: Converting Folate Precursor to Antimicrobial and Cytotoxic Agents

**DOI:** 10.3390/biom10010009

**Published:** 2019-12-19

**Authors:** Martin Krátký, Klára Konečná, Jiří Janoušek, Michaela Brablíková, Ondřej Janďourek, František Trejtnar, Jiřina Stolaříková, Jarmila Vinšová

**Affiliations:** 1 Department of Organic and Bioorganic Chemistry, Faculty of Pharmacy in Hradec Králové, Charles University, Akademika Heyrovského 1203, 500 05 Hradec Králové, Czech Republic; vinsova@faf.cuni.cz; 2 Department of Biological and Medical Sciences, Faculty of Pharmacy in Hradec Králové, Charles University, Akademika Heyrovského 1203, 500 05 Hradec Králové, Czech Republic; konecna@faf.cuni.cz (K.K.); jando6aa@faf.cuni.cz (O.J.); 3 Department of Pharmacology and Toxicology, Faculty of Pharmacy in Hradec Králové, Charles University, Akademika Heyrovského 1203, 500 05 Hradec Králové, Czech Republic; janousj2@faf.cuni.cz (J.J.); trejtnarf@faf.cuni.cz (F.T.); 4 Unipetrol Centre of Research and Education, 436 70 Litvínov-Záluží 1, Czech Republic; brablikova.michaela@gmail.com; 5 Laboratory for Mycobacterial Diagnostics and Tuberculosis, Regional Institute of Public Health in Ostrava, Partyzánské námĕstí 7, 702 00 Ostrava, Czech Republic; Jirina.Stolarikova@zuova.cz

**Keywords:** 4-aminobenzoic acid, antibacterial activity, antifungal activity, cytotoxicity, Schiff bases, synthesis, vitamin

## Abstract

4-aminobenzoic acid (PABA), an essential nutrient for many human pathogens, but dispensable for humans, and its derivatives have exhibited various biological activities. In this study, we combined two pharmacophores using a molecular hybridization approach: this vitamin-like molecule and various aromatic aldehydes, including salicylaldehydes and 5-nitrofurfural, via imine bond in one-step reaction. Resulting Schiff bases were screened as potential antimicrobial and cytotoxic agents. The simple chemical modification of non-toxic PABA resulted in constitution of antibacterial activity including inhibition of methicillin-resistant *Staphylococcus aureus* (minimum inhibitory concentrations, MIC, from 15.62 µM), moderate antimycobacterial activity (MIC ≥ 62.5 µM) and potent broad-spectrum antifungal properties (MIC of ≥ 7.81 µM). Some of the Schiff bases also exhibited notable cytotoxicity for cancer HepG2 cell line (IC_50_ ≥ 15.0 µM). Regarding aldehyde used for the derivatization of PABA, it is possible to tune up the particular activities and obtain derivatives with promising bioactivities.

## 1. Introduction

*Para*-aminobenzoic acid (4-aminobenzoic acid, *p*-aminobenzoic acid; PABA; **1**) is a well-known amino acid compound in biochemistry, medicinal chemistry and with many industrial applications.

PABA is synthesized and utilized as a substrate for the synthesis of folic acid in many bacterial species, yeasts and plants, i.e., playing a crucial role in this metabolic pathway. In addition to folate metabolism, PABA was identified as a coenzyme Q precursor and one of the factors required for microbial virulence. From traditional point of view, this compound belongs to the vitamins B group (vitamin B_x_ or B_10_). However, PABA is neither essential nor biosynthesized in mammals. It is produced by symbiotic bacteria, especially *Escherichia coli* and absorbed from dietary intake [1,2,3,4,5].

In contrast to its essentiality for some bacteria, when combined with several antibiotics, PABA applies a synergistic antibacterial potency for different bacterial strain including *Staphylococcus aureus* or *Pseudomonas aeruginosa*. PABA alone showed a direct antibacterial activity against *Listeria monocytogenes*, *Salmonella enteritidis* and *E. coli*. Low pH values potentiate this action. Analogous conclusions about adjunctive/synergistic activity were obtained for antivirotics [1,2,3,6]. It has also exhibited multiple antiviral and antioxidant actions. PABA and its derivatives have been utilized as active constituents in sunscreens to protect against UVB irradiation, i.e., scavengers of reactive oxygen species [1,2,3]. PABA demonstrated also properties of cytoprotective agents [7] and direct anticoagulants [2]. PABA-based diagnostic tests of gastrointestinal tract state have been developed [4,8]. The potassium 4-aminobenzoate is used for the treatment of mainly dermatologic disorders such as scleroderma, dermatomyositis and Peyronie’s disease [3]. In agriculture, PABA has displayed the promotion of resistance against plant bacterial and viral pathogens and plant growth modulator properties [4].

Nevertheless, absorption of PABA in the gastrointestinal tract represents an unfavourable feature [3]. The acid is absorbed through a passive process and it undergoes phase II biotransformation. PABA can be metabolized by either *N*-acetylation or in conjugation with glycine in the liver to produce 4-aminohippuric acid. Both these metabolic processes can take place to synthesize 4-acetamidohippuric acid. Then, PABA and its metabolites are almost completely excreted in the urine. PABA is generally considered non-toxic and well-tolerated. Side effects of systemic exposure are predominantly non-specific (nausea and skin rash), although hepatotoxicity may occur [8,9].

**Figure 1:**
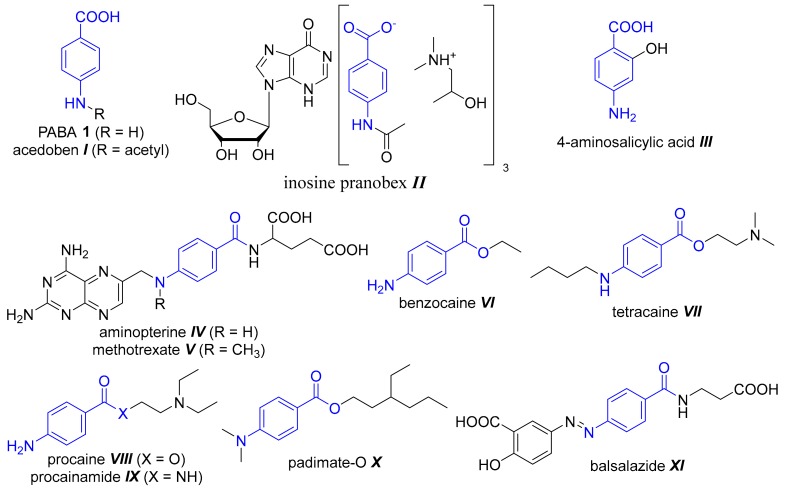
Examples of drugs containing the 4-aminobenzoic acid motif.

As mentioned above, the organisms biosynthesizing PABA obtain it from chorismate by a reaction catalysed by 4-amino-4-deoxychorismate synthase. This enzyme replaces the hydroxyl group at C4 by an amino group from chorismate, and it removes the pyruvic moiety subsequently to form 4-aminobenzoate [10]. The structure of PABA is highly specific and minimal structural alterations lead to inhibition of enzymes utilizing PABA or involved in subsequent metabolic steps. Illustratively, sulfonamides used for the treatment of many bacterial and protozoal infections act as structural analogues of PABA inhibiting dihydropteroate synthase competitively [11]. A natural metabolite of PABA, 4-acetamidobenzoic acid (acedoben, Figure 1, ***I***), is also able to interfere with the function of dihydropteroate synthase [12]. This molecule is one of the components of immunomodulating and antiviral drug inosine pranobex (Isoprinosine; Figure 1, ***II***) [13]. Antitubercular drug 4-aminosalicylic acid (Figure 1, ***III***) is utilized by dihydropteroate synthase instead of PABA and incorporated into the folate pathway to generate a hydroxyl dihydrofolate antimetabolite, which interferes with dihydrofolate reductase activity leading to the disruption of the biosynthesis of purines and thymidylate [11,14]. The 4-aminoaryl group is required for the biological functions and essentiality of PABA. Its chemical modification prevents its vitamin action and utilization.

In medicinal chemistry, a PABA-building block is used in design of drugs frequently and this scaffold can be found in many drugs with various therapeutic effects (Figure 1). We can distinguish “simple” PABA-derived drugs substituted at carboxyl and/or amino group basically, or more complex molecules with an additional substitution of benzene ring. Focusing on the first group mentioned, the carboxyl can be free, in salt form, or modified through ester or amide bond formation. The amino group is usually free, mono- and di-alkylated or incorporated into amide. For example, PABA-based drugs summarized in Figure 1 involve anticancer and cytotoxic folate antagonists (e.g., aminopterin ***IV***, methotrexate ***V***), alkylating agents, local anaesthetics (benzocaine ***VI***, tetracaine ***VII****,* procaine ***VIII***, etc.), antiarrhythmics (procainamide ***IX***), sunscreens (Padimate-O ***X***) or anti-inflammatory drugs (balsalazide ***XI***) [12].

**Figure 2:**
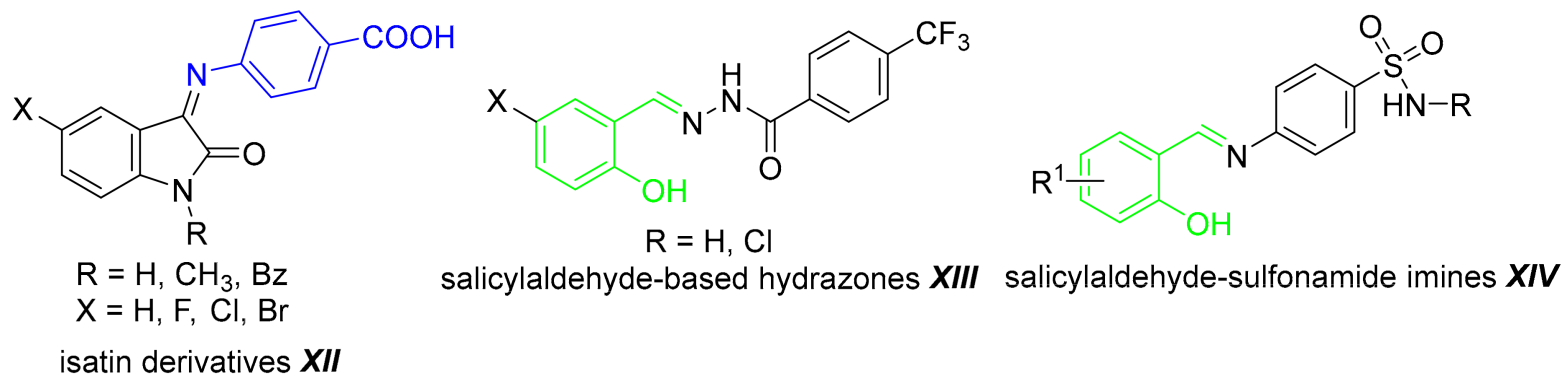
Examples of bioactive PABA (blue) and salicylaldehyde-derived (green) imines and hydrazones.

PABA hybrid compounds have been reported as potential antimicrobial agents. PABA has been linked with other bioactive scaffolds involving NH_2_ group via amide bond [15], as a secondary amine [1] or ureido moiety [16]. The derivatives of PABA showed improved radical scavenging properties and also enhanced toxicity for cancer cells [5]. PABA-ambazone salt exhibited improved antibacterial effect when compared to parent drugs [17]. Schiff bases with isatin (Figure 2, ***XII***) exhibited activity against various cancer cell lines [18]. Thus, the chemical modification of PABA provides a viable concept for modulation of its properties to obtain both antimicrobial and cytotoxic compounds.

Our present approach is focused on the synthesis of Schiff bases of PABA and aldehydes, mainly salicylaldehydes, and their evaluation as potential antimicrobial and cytotoxic agents. These fused compounds combine two pharmacophores through the imine bond. Previously, we have applied molecular hybridization approach successfully. Illustratively, salicylaldehyde-based imines and hydrazones derived from known bioactive scaffolds (Figure 2, ***XIII***–***XIV***) have exhibited a significant antimicrobial activity [19,20,21]. We have described that a potent antimicrobial activity is associated only with the salicylidene moiety and its isomers are substantially less active [21,22]. That is why we designed predominantly 2-substituted benzylidene scaffold for this study. The modification of the amino group can result in altered vitamin function in susceptible strains and novel derivatives with an enhanced lipophilicity may promote limited absorption and intracellular accumulation of PABA via passive diffusion.

## 2. Materials and Methods

### 2.1. Chemistry 

#### 2.1.1. General

All the reagents and solvents were purchased from Sigma-Aldrich (Darmstadt, Germany) or Penta Chemicals (Prague, Czech Republic) and they were used as received. The reactions and the purity of the products were monitored by thin-layer chromatography (TLC) using a mixture with a ratio of dichloromethane (DCM) to methanol (MeOH) of 4:1 (*v*/*v*) as the eluent. TLC plates were coated with 0.2 mm Merck 60 F254 silica gel (Merck Millipore, Darmstadt, Germany) with UV detection (254 nm). The melting points were determined on a Büchi Melting Point B-540 apparatus (BÜCHI, Flawil, Switzerland) using open capillaries. The reported values are uncorrected. Infrared spectra were recorded on a FT-IR spectrometer using ATR-Ge method (Nicolet 6700 FT-IR, Thermo Fisher Scientific, Waltham, MA, USA) in the range of 650–4000 cm^−1^. The NMR spectra were measured in DMSO-*d_6_* at ambient temperature using a Varian V NMR S500 instrument (500 MHz for ^1^H and 126 MHz for ^13^C; Varian Comp. Palo Alto, CA, USA). The chemical shifts δ are given in ppm and were referred indirectly to tetramethylsilane via signals of DMSO-*d_6_* (2.49 for ^1^H and 39.7 for ^13^C spectra). The coupling constants (*J*) are reported in Hz. Elemental analysis was performed on Vario MICRO Cube Element Analyzer (Elementar Analysensysteme, Hanau, Germany). Both calculated and found values are given as percentages. 

#### 2.1.2. Synthesis of PABA-Derived Schiff Bases **1a**–**1t**

4-aminobenzoic acid (137.1 mg; 1 mmol) was dissolved in 7 mL of MeOH and then appropriate aldehyde (1.1 mmol) was added in one portion. The reaction mixture was heated under reflux for 3 h, then let stir at room temperature for 12 h. After this time, the reaction mixture was stored at −20 °C for 1 h. The resulted precipitate was filtered off and washed by cold diethyl ether (DEE) thoroughly. The products were crystallized from MeOH to obtain pure Schiff bases if necessary. Alternatively, Schiff bases can be prepared using DEE as a solvent instead of MeOH. In this case, the reaction mixture was stirred for 72 h at room temperature. The practical yields of both methods were comparable.

Toluene-4-sulfonic acid (as monohydrate; 19.0 mg; 0.1 of equivalents) was applied as an acidic catalyst in the synthesis of two products (**1f**, **1t**), when the uncatalyzed reaction provided low or almost no yields.

The identity of the known compounds was established using NMR and IR spectroscopy by the comparison with previously reported data. Additionally, their purity was checked by melting points measurement and elemental analysis. The compounds were considered pure if they agree within ± 0.4% with theoretical values.

*4-[(2-Hydroxybenzylidene)amino]benzoic acid* (**1a**). Yellow solid; yield 92%; mp 265.5–268 °C (lit. [23] 276–278 °C). IR: 1690 (COOH), 1608 (-HC=N-) cm^−1^. ^1^H-NMR (500 MHz, DMSO-*d*_6_): δ 12.95 (1H, s, COOH), 12.71 (1H, s, OH), 8.99 (1H, s, CH=N), 8.03–7.99 (2H, m, H2, H6), 7.69 (1H, dd, *J* = 7.6 Hz, 1.8 Hz, H6′), 7.51–7.42 (3H, m, H3, H5, H4′), 7.02–6.96 (2H, m, H3′, H5′). ^13^C-NMR (126 MHz, DMSO-*d_6_*): δ 167.06, 164.99, 160.49, 152.34, 134.02, 132.80, 130.92, 128.11, 121.71, 121.22, 119.50, 115.88. Anal. Calcd. for C_14_H_11_NO_3_ (241.25): C, 69.70; H, 4.60; N, 5.81. Found: C, 69.79; H, 4.74; N, 5.70.

*4-[(5-Fluoro-2-hydroxybenzylidene)amino]benzoic acid* (**1b**). Orange solid; yield 90%; mp 277–278.5 °C. IR: 1678 (COOH), 1570 (-HC=N-) cm^−1^. ^1^H-NMR (500 MHz, DMSO-*d*_6_): δ 12.92 (1H, s, COOH), 12.28 (1H, s, OH), 8.94 (1H, s, CH=N), 8.03–7.99 (2H, m, H2, H6), 7.54 (1H, dd, *J* = 8.9 Hz, 3.2 Hz, H6′), 7.48–7.43 (2H, m, H3, H5), 7.30 (ddd, *J* = 9.1 Hz, 8.3 Hz, 3.2 Hz, H4′), 7.00 (1H, dd, *J* = 9.1 Hz, 4.5 Hz, H3′). ^13^C-NMR (126 MHz, DMSO-*d_6_*): δ 167.05, 163.30 (d, *J* = 2.7 Hz), 156.64, 155.20 (d, *J* = 234.9 Hz), 152.46, 130.94, 129.15, 121.70, 120.97 (d, *J* = 23.5 Hz), 119.94 (d, *J* = 7.5 Hz), 118.34 (d, *J* = 7.6 Hz), 116.90 (d, *J* = 23.4 Hz). Anal. Calcd. for C_14_H_10_FNO_3_ (259.24): C, 64.87; H, 3.89; N, 5.40. Found: C, 64.69; H, 3.71; N, 5.64.

*4-[(5-Chloro-2-hydroxybenzylidene)amino]benzoic acid* (**1c**). Yellow solid; yield 91%; mp 302.5–304 °C (lit. [24] 230–232 °C). IR: 1681 (COOH), 1599 (-HC=N-) cm^−1^. ^1^H-NMR (500 MHz, DMSO-*d*_6_): δ 12.97 (1H, s, COOH), 12.60 (1H, s, OH), 8.95 (1H, s, CH=N), 8.04–7.99 (2H, m, H2, H6), 7.78 (1H, d, *J* = 2.7 Hz, H6′), 7.48–7.43 (3H, m, H3, H5, H4′), 7.01 (1H, d, *J* = 8.8 Hz, H3′). ^13^C-NMR (126 MHz, DMSO-*d_6_*): δ 167.02, 163.24, 159.05, 152.27, 133.42, 131.01, 130.93, 129.21, 122.93, 121.73, 120.89, 118.93. Anal. Calcd. for C_14_H_10_ClNO_3_ (275.69): C, 60.99; H, 3.66; N, 5.08. Found: C, 61.11; H, 3.72; N, 5.16.

*4-[(5-Bromo-2-hydroxybenzylidene)amino]benzoic acid* (**1d**). Orange solid; yield 87%; mp 281.5–284 °C (lit. [25] 282 °C). IR: 1681 (COOH), 1596 (-HC=N-) cm^−1^. ^1^H-NMR (500 MHz, DMSO-*d*_6_): δ 12.92 (1H, s, COOH), 12.62 (1H, s, OH), 8.94 (1H, s, CH=N), 8.03–7.99 (2H, m, H2, H6), 7.90 (1H, d, *J* = 2.6 Hz, H6′), 7.57 (1H, dd, *J* = 8.8 Hz, 2.6 Hz, H4′), 7.48–7.44 (2H, m, H3, H5), 6.96 (1H, d, *J* = 8.8 Hz, H3′). ^13^C-NMR (126 MHz, DMSO-*d_6_*): δ 167.04, 163.18, 159.47, 152.27, 136.20, 134.00, 130.95, 129.22, 121.75, 121.52, 119.36, 110.31. Anal. Calcd. for C_14_H_10_BrNO_3_ (320.14): C, 52.52; H, 3.15; N, 4.38. Found: C, 52.25; H, 3.04; N, 4.44.

*4-[(2-Hydroxy-5-iodobenzylidene)amino]benzoic acid* (**1e**). Orange solid; yield 96%; mp 302–304 °C. IR: 1680 (COOH), 1593 (-HC=N-) cm^−1^. ^1^H-NMR (500 MHz, DMSO-*d*_6_): δ 12.96 (1H, s, COOH), 12.64 (1H, s, OH), 8.92 (1H, s, CH=N), 8.04 (1H, d, *J* = 2.3 Hz, H6′), 8.03–7.99 (2H, m, H2, H6), 7.70 (1H, dd, *J* = 8.7 Hz, 2.3 Hz, H4′), 7.48–7.44 (2H, m, H3, H5), 6.84 (1H, d, *J* = 8.7 Hz, H3′). ^13^C-NMR (126 MHz, DMSO-*d_6_*): δ 167.02, 163.20, 160.00, 152.26, 141.81, 140.04, 130.93, 129.16, 122.12, 121.74, 119.64, 81.08. Anal. Calcd. for C_14_H_10_INO_3_ (367.14): C, 45.80; H, 2.75; N, 3.82. Found: C, 46.02; H, 3.00; N, 3.55.

*4-[(2-Hydroxy-5-nitrobenzylidene)amino]benzoic acid* (**1f**). Yellowish orange solid; yield 81%; mp 311–313 °C (decomp.). IR: 1693 (COOH), 1597 (-HC=N-) cm^−1^. ^1^H-NMR (500 MHz, DMSO-*d*_6_): δ 13.78 (1H, s, COOH), 12.87 (1H, s, OH), 9.14 (1H, s, CH=N), 8.69 (1H, d, *J* = 2.9 Hz, H6′), 8.28 (1H, dd, *J* = 9.2 Hz, 2.9 Hz, H4′), 8.05–8.01 (2H, m, H2, H6), 7.54–7.50 (3H, m, H3, H5), 7.14 (1H, d, *J* = 9.2 Hz, H3′). ^13^C-NMR (126 MHz, DMSO-*d_6_*): δ 166.95, 166.31, 162.66, 151.24, 139.63, 130.95, 129.56, 128.90, 128.16, 121.74, 119.21, 118.36. Anal. Calcd. for C_14_H_10_N_2_O_5_ (286.24): C, 58.75; H, 3.52; N, 9.79. Found: C, 58.86; H, 3.33; N, 10.02.

*4-[(2-Hydroxy-5-methylbenzylidene)amino]benzoic acid* (**1g**). Yellow solid; yield 92%; mp 271–273 °C (lit. [26] 258 °C). IR: 1681 (COOH), 1574 (-HC=N-) cm^−1^. ^1^H-NMR (500 MHz, DMSO-*d*_6_): δ 12.94 (1H, s, COOH), 12.41 (1H, s, OH), 8.91 (1H, s, CH=N), 8.03–7.99 (2H, m, H2, H6), 7.50–7.43 (3H, m, H3, H5, H6′), 7.25 (1H, dd, *J* = 8.4 Hz, 2.3 Hz, H4′), 6.88 (1H, d, *J* = 8.3 Hz, H3′), 2.27 (3H, s, CH_3_). ^13^C-NMR (126 MHz, DMSO-*d_6_*): δ 167.05, 164.74, 158.33, 152.52, 134.78, 132.42, 130.90, 128.87, 128.04, 121.63, 119.18, 116.72, 20.09. Anal. Calcd. for C_15_H_13_NO_3_ (255.27): C, 70.58; H, 5.13; N, 5.49. Found: C, 70.81; H, 5.01; N, 5.57.

*4-[(2-Hydroxy-5-methoxybenzylidene)amino]benzoic acid* (**1h**) [27]. Brownish yellow solid; yield 92%; mp 247.5–249.5 °C (decomp.). IR: 1677 (COOH), 1569 (-HC=N-) cm^−1^. ^1^H-NMR (500 MHz, DMSO-*d*_6_): δ 12.93 (1H, s, COOH), 12.04 (1H, s, OH), 8.94 (1H, s, CH=N), 8.03–7.99 (2H, m, H2, H6), 7.46–7.43 (2H, m, H3, H5), 7.28 (1H, d, *J* = 3.2 Hz, H6′), 7.06 (1H, dd, *J* = 9.0 Hz, 3.2 Hz, H4′), 6.92 (1H, d, *J* = 8.9 Hz, H3′), 3.75 (3H, s, OCH_3_). ^13^C-NMR (126 MHz, DMSO-*d_6_*): δ 167.06, 164.31, 154.58, 152.70, 152.15, 130.92, 128.89, 121.63, 121.36, 119.44, 117.81, 115.01, 55.73. Anal. Calcd. for C_15_H_13_NO_4_ (271.27): C, 66.41; H, 4.83; N, 5.16. Found: C, 66.35; H, 4.69; N, 5.01.

*4-[(2,5-Dihydroxybenzylidene)amino]benzoic acid* (**1i**) [28]. Orange solid; yield 81%; mp 278–280 °C (decomp.). IR: 1679 (COOH), 1574 (-HC=N-) cm^−1^. ^1^H-NMR (500 MHz, DMSO-*d*_6_): δ 12.89 (1H, s, COOH), 11.87 (1H, s, 2-OH), 9.13 (1H, s, 5-OH), 8.87 (1H, s, CH=N), 8.02–7.98 (2H, m, H2, H6), 7.46–7.43 (2H, m, H3, H5), 7.08 (1H, d, *J* = 3.0 Hz, H6′), 6.89 (1H, dd, *J* = 8.8 Hz, 3.0 Hz, H4′), 6.81 (1H, d, *J* = 8.8 Hz, H3′). ^13^C-NMR (126 MHz, DMSO-*d_6_*): δ 167.08, 164.50, 153.35, 152.78, 149.92, 130.88, 128.78, 121.95, 121.66, 119.54, 117.52, 116.89. Anal. Calcd. for C_14_H_11_NO_4_ (257.07): C, 65.37; H, 4.31; N, 5.45. Found: C, 65.55; H, 4.40; N, 5.62.

*4-{[5-(Tert-butyl)-2-hydroxybenzylidene]amino}benzoic acid* (**1j**). Orange solid; yield 85%; mp 270–272 °C. IR: 1682 (COOH), 1569 (-HC=N-) cm^−1^. ^1^H-NMR (500 MHz, DMSO-*d*_6_): δ 12.94 (1H, s, COOH), 12.51 (1H, s, OH), 8.99 (1H, s, CH=N), 8.03–7.99 (2H, m, H2, H6), 7.70 (1H, d, *J* = 2.6 Hz, H6′), 7.50–7.46 (3H, m, H3, H5, H4′), 6.92 (1H, d, *J* = 8.7 Hz, H3′), 1.28 (9H, s, CH_3_). ^13^C-NMR (126 MHz, DMSO-*d_6_*): δ 167.08, 165.40, 158.35, 152.52, 141.66, 131.38, 130.92, 129.04, 128.86, 121.68, 118.75, 116.51, 34.01, 31.35. Anal. Calcd. for C_18_H_19_NO_3_ (297.35): C, 72.71; H, 6.44; N, 4.71. Found: C, 72.57; H, 6.58; N, 4.50.

*4-[(2-Chloro-6-hydroxybenzylidene)amino]benzoic acid* (**1k**). Orange solid; yield 94%; mp 279–281 °C. IR: 1681 (COOH), 1596 (-HC=N-) cm^−1^. ^1^H-NMR (500 MHz, DMSO-*d*_6_): δ 14.02 (1H, s, COOH), 13.03 (1H, s, OH), 9.17 (1H, s, CH=N), 8.05–8.01 (2H, m, H2, H6), 7.57–7.53 (2H, m, H3, H5), 7.45 (1H, t, *J* = 8.2 Hz, H4′), 7.07 (1H, dd, *J* = 7.9 Hz, 1.1 Hz, H3′), 6.98 (1H, dd, *J* = 8.4 Hz, 1.0 Hz, H5′). ^13^C-NMR (126 MHz, DMSO-*d_6_*): δ 166.95, 162.79, 161.81, 151.04, 135.83, 134.98, 130.99, 129.65, 121.93, 120.46, 116.83, 115.66. Anal. Calcd. for C_14_H_10_ClNO_3_ (275.69): C, 60.99; H, 3.66; N, 5.08. Found: C, 60.84; H, 3.74; N, 5.01.

*4-[(4-Chloro-2-hydroxybenzylidene)amino]benzoic acid* (**1l**). Yellowish orange solid; yield 90%; mp 304–307 °C (decomp.). IR: 1674 (COOH), 1596 (-HC=N-) cm^−1^. ^1^H-NMR (500 MHz, DMSO-*d*_6_): δ 13.05 (1H, s, COOH), 12.72 (1H, s, OH), 8.99 (1H, s, CH=N), 8.04–7.99 (2H, m, H2, H6), 7.72 (1H, d, *J* = 8.1 Hz, H6′), 7.48–7.43 (2H, m, H3, H5), 7.07–7.03 (2H, m, H3′, H5′). ^13^C-NMR (126 MHz, DMSO-*d_6_*): δ 167.01, 163.73, 161.30, 152.01, 138.18, 133.89, 130.90, 129.13, 121.70, 119.79, 118.64, 116.76. Anal. Calcd. for C_14_H_10_ClNO_3_ (275.69): C, 60.99; H, 3.66; N, 5.08. Found: C, 60.77; H, 3.65; N, 5.20.

*4-[(3-Chloro-2-hydroxybenzylidene)amino]benzoic acid* (**1m**). Yellow solid; yield 85%; mp 296–298 °C. IR: 1689 (COOH), 1596 (-CH=N-) cm^−1^. ^1^H-NMR (500 MHz, DMSO-*d*_6_): δ 13.99 (1H, s, COOH), 13.01 (1H, s, OH), 9.08 (1H, s, CH=N), 8.05–8.01 (2H, m, H2, H6), 7.65 (1H, dd, *J* = 7.8 Hz, 1.6 Hz, H6′), 7.61 (1H, dd, *J* = 7.9 Hz, 1.5 Hz, H4′), 7.57–7.54 (2H, m, H3, H5), 7.01 (1H, t, *J* = 7.8 Hz, H5′). ^13^C-NMR (126 MHz, DMSO-*d_6_*): δ 166.95, 165.17, 156.69, 150.75, 133.77, 132.22, 130.92, 129.52, 121.87, 120.56, 120.19, 119.85. Anal. Calcd. for C_14_H_10_ClNO_3_ (275.69): C, 60.99; H, 3.66; N, 5.08. Found: C, 61.09; H, 3.79; N, 5.30.

*4-[(3,5-Dichloro-2-hydroxybenzylidene)amino]benzoic acid* (**1n**). Orange solid; yield 89%; mp 317.5–319 °C (lit. [29] 300 °C). IR: 1689 (COOH), 1597 (-CH=N-) cm^−1^. ^1^H-NMR (500 MHz, DMSO-*d*_6_): δ 14.02 (1H, s, COOH), 13.03 (1H, s, OH), 9.04 (1H, s, CH=N), 8.05-8.01 (2H, m, H2, H6), 7.75–7.73 (2H, m, H4′, H6′), 7.56–7.53 (2H, m, H3, H5). ^13^C-NMR (126 MHz, DMSO-*d_6_*): δ 166.90, 164.07, 155.94, 150.30, 132.83, 131.06, 130.96, 129.83, 122.47, 121.93, 121.88, 120.66. Anal. Calcd. for C_14_H_9_Cl_2_NO_3_ (309.00): C, 54.22; H, 2.93; N, 4.52. Found: C, 54.10; H, 3.11; N, 4.36.

*4-[(3-Bromo-5-chloro-2-hydroxybenzylidene)amino]benzoic acid* (**1o**). Orangish solid; yield 92%; mp > 300 °C (decomp.). IR: 1685 (COOH), 1595 (-CH=N-) cm^−1^. ^1^H-NMR (500 MHz, DMSO-*d*_6_): δ 14.05 (1H, s, COOH), 13.03 (1H, s, OH), 9.02 (1H, s, CH=N), 8.05–8.02 (2H, m, H2, H6), 7.86 (1H, d, *J* = 2.5 Hz, H4′), 7.77 (1H, d, *J* = 2.6 Hz, H6′), 7.57–7.53 (2H, m, H3, H5). ^13^C-NMR (126 MHz, DMSO-*d_6_*): δ 166.88, 164.08, 156.90, 150.16, 135.60, 131.76, 130.95, 129.82, 122.84, 121.90, 120.38, 111.36. Anal. Calcd. for C_14_H_9_BrClNO_3_ (352.95): C, 47.42; H, 2.56; N, 3.95. Found: C, 47.55; H, 2.70; N, 4.13.

*4-[(5-Chloro-2-hydroxy-3-iodobenzylidene)amino]benzoic acid* (**1p**). Reddish orange solid; yield 84%; mp 290 °C (decomp). IR: 1671 (COOH), 1589 (-CH=N-) cm^−1^. ^1^H-NMR (500 MHz, DMSO-*d*_6_): δ 14.04 (1H, s, COOH), 13.03 (1H, s, OH), 8.96 (1H, s, CH=N), 8.05–8.02 (2H, m, H2, H6), 7.97 (1H, d, *J* = 2.6 Hz, H4′), 7.78 (1H, d, *J* = 2.6 Hz, H6′), 7.58–7.53 (2H, m, H3, H5). ^13^C-NMR (126 MHz, DMSO-*d_6_*): δ 166.90, 164.09, 159.36, 150.17, 141.24, 132.50, 130.96, 129.77, 123.32, 121.94, 119.23, 87.53. Anal. Calcd. for C_14_H_9_ClINO_3_ (400.93): C, 41.87; H, 2.26; N, 3.49. Found: C, 42.11; H, 2.39; N, 3.31.

*4-[(2-Hydroxy-3,5-diiodobenzylidene)amino]benzoic acid* (**1q**). Red solid; yield 95%; mp 289–291 °C (decomp.). IR: 1667 (COOH), 1583 (-CH=N-) cm^−1^. ^1^H-NMR (500 MHz, DMSO-*d*_6_): δ 14.08 (1H, s, COOH), 13.02 (1H, s, OH), 8.94 (1H, s, CH=N), 8.16 (1H, d, *J* = 2.1 Hz, H4′), 8.05–8.00 (3H, m, H2, H6, H6′), 7.57–7.53 (2H, m, H3, H5). ^13^C-NMR (126 MHz, DMSO-*d_6_*): δ 166.90, 163.95, 160.22, 150.14, 148.93, 141.47, 130.95, 129.72, 121.91, 120.77, 88.51, 81.51. Anal. Calcd. for C_14_H_9_I_2_NO_3_ (492.87): C, 34.11; H, 1.84; N, 2.84. Found: C, 33.87; H, 2.00; N, 2.87.

*4-{[(5-Nitrofuran-2-yl)methylene]amino}benzoic acid* (**1r**). Yellow solid; yield 80%; mp 233–235 °C (decomp.) (lit. [30] 234 °C decomp.). IR: 1673 (COOH), 1591 (-CH=N-) cm^−1^. ^1^H-NMR (500 MHz, DMSO-*d*_6_): δ 12.95 (1H, s, COOH), 8.64 (1H, s, CH=N), 8.03–7.98 (2H, m, H2, H6), 7.83 (1H, d, *J* = 3.9 Hz, furan H4′), 7.48 (1H, d, *J* = 3.9 Hz, furan H3′), 7.43–7.39 (2H, m, H3, H5). ^13^C-NMR (126 MHz, DMSO-*d_6_*): δ 167.00, 153.92, 152.74, 152.47, 149.99, 130.84, 129.37, 121.53, 119.15, 114.20. Anal. Calcd. for C_12_H_8_N_2_O_5_ (260.04): C, 55.39; H, 3.10; N, 10.77. Found: C, 55.60; H, 2.94; N, 10.89.

*4-[(2-Methoxybenzylidene)amino]benzoic acid* (**1s**). White solid; yield 95%; mp 231.5–234 °C (lit. [24] 220–221 °C). IR: 1683 (COOH), 1593 (-HC=N-) cm^−1^. ^1^H-NMR (500 MHz, DMSO-*d*_6_): δ 12.85 (1H, s, COOH), 8.84 (1H, s, CH=N), 8.02 (1H, dd, *J* = 7.7 Hz, 1.8 Hz, H6′), 7.99–7.95 (2H, m, H2, H6), 7.54 (1H, ddd, *J* = 8.8 Hz, 7.2 Hz, 1.8 Hz, H4′), 7.28–7.23 (2H, m, H3, H5), 7.17 (1H, d, *J* = 8.4 Hz, H3′), 7.07 (1H, t, *J* = 7.5 Hz, H5′). ^13^C-NMR (126 MHz, DMSO-*d_6_*): δ 167.21, 159.72, 157.26, 156.24, 133.92, 130.84, 127.99, 127.21, 123.68, 121.09, 120.91, 112.30, 56.01. Anal. Calcd. for C_15_H_13_NO_3_ (255.09): C, 70.58; H, 5.13; N, 5.49. Found: C, 70.44; H, 5.02; N, 5.60.

*4-[(4-Hydroxybenzylidene)amino]benzoic acid* (**1t**). Yellowish brown solid; yield 80%; mp 251–253 °C (lit. [31] 254–255 °C). IR: 1681 (COOH), 1599 (-HC=N-) cm^−1^. ^1^H-NMR (500 MHz, DMSO-*d*_6_): δ 10.45 (1H, s, COOH), 9.79 (1H, s, OH), 8.65 (1H, s, CH=N), 8.00–7.97 (2H, m, H2, H6), 7.79–7.74 (2H, m, H2′, H6′), 7.38–7.34 (2H, m, H3, H5). 6.96–6.93 (2H, m, H3′, H5′). ^13^C-NMR (126 MHz, DMSO-*d_6_*): δ 167.59, 162.02, 161.41, 156.39, 131.39, 130.86, 128.03, 127.65, 121.12, 116.27. Anal. Calcd. for C_14_H_11_NO_3_ (241.25): C, 69.70; H, 4.60; N, 5.81. Found: C, 69.66; H, 4.51; N, 5.97.

#### 2.1.3. Synthesis of **1u**

4-aminobenzoic acid (137.1 mg; 1 mmol) was dissolved in 7 mL of MeOH, followed by addition of salicylaldehyde (117 µL; 1.1 mmol), sodium cyanoborohydride (62.8 mg; 1 mmol) and finally glacial acetic acid (100 µL). The mixture was stirred for 24 h at room temperature. After this time, the reaction mixture was diluted with distilled water and stirred for additional 1 h. The resulted precipitate was filtered off and washed by water. The product was crystallized from ethyl acetate/*n*-hexane to obtain pure **1u**. 

*4-[(2-Hydroxybenzyl)amino]benzoic acid* (**1u**). Greyish solid; yield 74%; mp 198–199.5 °C (lit. [32] 203–204 °C). IR: 3277, 1670 (COOH) cm^−1^. ^1^H-NMR (500 MHz, DMSO-*d*_6_): δ 11.95 (1H, s, COOH), 9.56 (1H, s, OH), 7.64 (2H, d, *J* = 8.7 Hz, H2, H6), 7.14 (1H, dd, *J* = 7.6 Hz, 2.7 Hz, H6′), 7.05 (1H, td, *J* = 7.6 Hz, 1.7 Hz, H4′), 6.85-6.79 (2H, m, NH, H3′), 6.73 (1H, td, *J* = 7.4 Hz, 1.1 Hz, H5′), 6.60-6.56 (2H, m, H3, H5), 4.24 (2H, d, *J* = 5.8 Hz, CH_2_). ^13^C-NMR (126 MHz, DMSO-*d_6_*): δ 167.68, 155.21, 152.80, 131.26, 128.39, 127.93, 125.09, 119.00, 117.06, 115.11, 111.14, 41.03. Anal. Calcd. for C_14_H_13_NO_3_ (243.09): C, 69.12; H, 5.39; N, 5.76. Found: C, 69.21; H, 5.51; N, 5.49.

### 2.2. Pharmacology

#### 2.2.1. Antibacterial Activity

For the purpose of screening for antibacterial activity of the derivatives four Gram-positive and four Gram-negative strains of clinical importance were used, namely: *Staphylococcus aureus* ATCC 29213, CCM 4223, methicillin-resistant *Staphylococcus aureus* ATCC 43300, CCM 4750 (MRSA), *Staphylococcus epidermidis* H 6966/08 (clinical isolate), *Enterococcus faecalis* ATCC 29212, CCM 4224; *Escherichia coli* ATCC 25922, CCM 3954, *Klebsiella pneumoniae* D 11750/08 (clinical isolate), extended-spectrum β-lactamase positive *Klebsiella pneumoniae* J 14368/08 (clinical isolate), and *Pseudomonas aeruginosa* ATCC 27853, CCM 3955. These strains were obtained from the Czech Collection of Microorganisms (CCM, Brno, Czech Republic) or they are clinical isolates from the Department of Clinical Microbiology, University Hospital in Hradec Králové, Czech Republic.

The microdilution broth method was performed according to EUCAST (The European Committee on Antimicrobial Susceptibility Testing) instructions with slight modifications [33]. Briefly, the cultivation was done in Cation-adjusted Mueller-Hinton broth (CAMHB, M-H 2 Broth, Sigma-Aldrich) at 35 ± 2 °C. Tested compounds were dissolved in DMSO (Sigma-Aldrich, St. Louis, MO, USA) to produce stock solutions. The final concentration of DMSO in the testing medium did not exceed 1% (*v*/*v*) of the total solution composition and did not affect the growth of bacteria. Positive (microbe solely), negative (cultivation medium and DMSO) controls and internal quality standards were involved in each assay. Antibacterial activity is expressed as minimum inhibitory concentration (MIC, reported in µM) after 24 and 48 h of static incubation in the dark and humidified atmosphere at 35 ± 2 °C. The experiments were performed in duplicates. For the results to be valid, the difference in MIC determined from two parallel measurements must not be greater than one step on the dilution scale. The results were analysed by visual inspection.

Parent PABA as well as topical antibiotics bacitracin (active especially against Gram-positive species including MRSA) and neomycin (an aminoglycoside active predominantly against Gram-negative strains) were involved as the reference drugs. Standard antibiotics (ciprofloxacin, gentamicin; data not shown) and internal quality control/reference strains are routinely included in parallel testing-basic screening of antibacterial activity.

#### 2.2.2. Antimycobacterial Activity

The in vitro antimycobacterial activity against *Mycobacterium tuberculosis* H_37_Rv (331/88; *Mtb.*) and three strains of nontuberculous mycobacteria (*Mycobacterium avium* 330/88 [resistant to isoniazid, rifamycines, ofloxacin, and ethambutol], *Mycobacterium kansasii* 235/80 from the Czech National Collection of Type Cultures, and a clinical isolate of *M. kansasii* 6509/96) was evaluated using a previously reported method [34]. The micromethod for the determination of MIC was used involving the Šula’s semisynthetic medium (SEVAC, Prague, Czech Republic). The investigated compounds were added to the medium as solutions in DMSO; the final volume contained 1.0% DMSO (*v*/*v*). The following concentrations were used: 1000, 500, 250, 125, 62.5, 32, 16, 8, 4, 2 and 1 μM. The MIC were determined after incubation at 37 °C for 14 and 21 days, for *M. kansasii* strains additionally for 7 days. MIC (in μM) was the lowest concentration at which the complete inhibition of mycobacterial growth occurred. First-line oral antitubercular drug isoniazid (INH) and parent PABA were involved as reference compounds.

#### 2.2.3. Antifungal Activity

The antifungal properties were evaluated against five yeast strains, namely: *Candida albicans* ATCC 44859, *Candida tropicalis* 156, *Candida krusei* E28, *Candida glabrata* 20/I, *Trichosporon asahii* 1188 and three strains of filamentous fungi: *Aspergillus fumigatus* 231, *Lichtheimia corymbifera* 272, and *Trichophyton interdigitale* 445. A microdilution broth method was performed according to EUCAST II, III instructions with slight modifications [35,36]. Briefly, tested compounds were dissolved in DMSO and diluted in a twofold manner with RPMI-1640 medium with l-glutamine, supplemented with 2% glucose (*w*/*v*) and buffered to pH 7.0 with 3 morpholinopropane-1-sulfonic acid (all components were purchased from Sigma-Aldrich, USA). The final concentration of DMSO in the tested medium did not exceed 1% (*v*/*v*) of the total solution composition, and it was confirmed that this concentration did not inhibit the fungal growth. Static incubation was performed in the dark and humidified atmosphere, at 35 ± 2 °C for 24 and 48 h (72 and 120 h for *Trichophyton interdigitale*). Positive controls consisted of test microbe solely, while negative controls consisted of medium and DMSO. Internal quality control was included too. Visual inspection was used for MIC endpoints evaluation. The experiments were conducted in duplicates. For the results to be valid, the difference in MIC determined from two parallel measurements must not be greater than one step on the dilution scale.

PABA and triazole antimycotic drug fluconazole were involved as the reference compounds. MIC values of fluconazole mean MIC_50_ values.

#### 2.2.4. Cytotoxicity

HepG2, human hepatocellular carcinoma cells (ATCC HB-8065; passage 5–8) purchased from Health Protection Agency Culture Collections (ECACC, Salisbury, UK) were cultured in Minimum Essentials Eagle Medium supplemented with 10% fetal bovine serum, 1% l-glutamine solution and non-essential amino acid solution (Sigma-Aldrich, St. Louis, MO, USA) in a humidified atmosphere containing 5% CO_2_ at 37 °C. For subculturing, the cells were harvested after trypsin/EDTA (Sigma-Aldrich, St. Louis, USA) treatment at 37 °C. To evaluate cytotoxicity, the cells treated with the tested substances were used as experimental groups whereas untreated HepG2 cells served as control groups.

The cells were seeded in a density of 10 000 cells per well in a 96-well plate. The following day the cells were treated with each of the tested substances dissolved in DMSO (maximal incubation concentration of DMSO was 1% *v*/*v*). The tested substances were prepared at different incubation concentrations in triplicates according to their solubility. Simultaneously, the controls representing 100% cell viability, 0% cell viability (the cells treated with 10% DMSO), no cell control and vehiculum controls were also involved in triplicates. After 24 h incubation in a humidified atmosphere containing 5% CO_2_ at 37 °C, the reagent from the kit CellTiter 96 AQueous One Solution Cell Proliferation Assay (CellTiter 96; Promega, Fitchburg, WI, USA) was added. After 2 h incubation at 37 °C absorbance of samples was recorded at 490 nm (TECAN, Infinita M200, Grödig, Austria). A standard toxicological parameter IC_50_ was calculated by nonlinear regression from a semilogarithmic plot of incubation concentration versus percentage of absorbance relative to untreated controls using GraphPad Prism software (version 6; GraphPad Software, Inc., La Jolla, CA, USA). Results of the experiments are presented as inhibitory concentration which reduces the viability of the cell population to 50% from the maximal viability (IC_50_).

Parent PABA and a cytotoxic/anticancer drugs tamoxifen (as tamoxifen citrate) and cisplatin were involved as the reference drugs.

## 3. Results and Discussion

### 3.1. Chemistry 

**Scheme 1:**
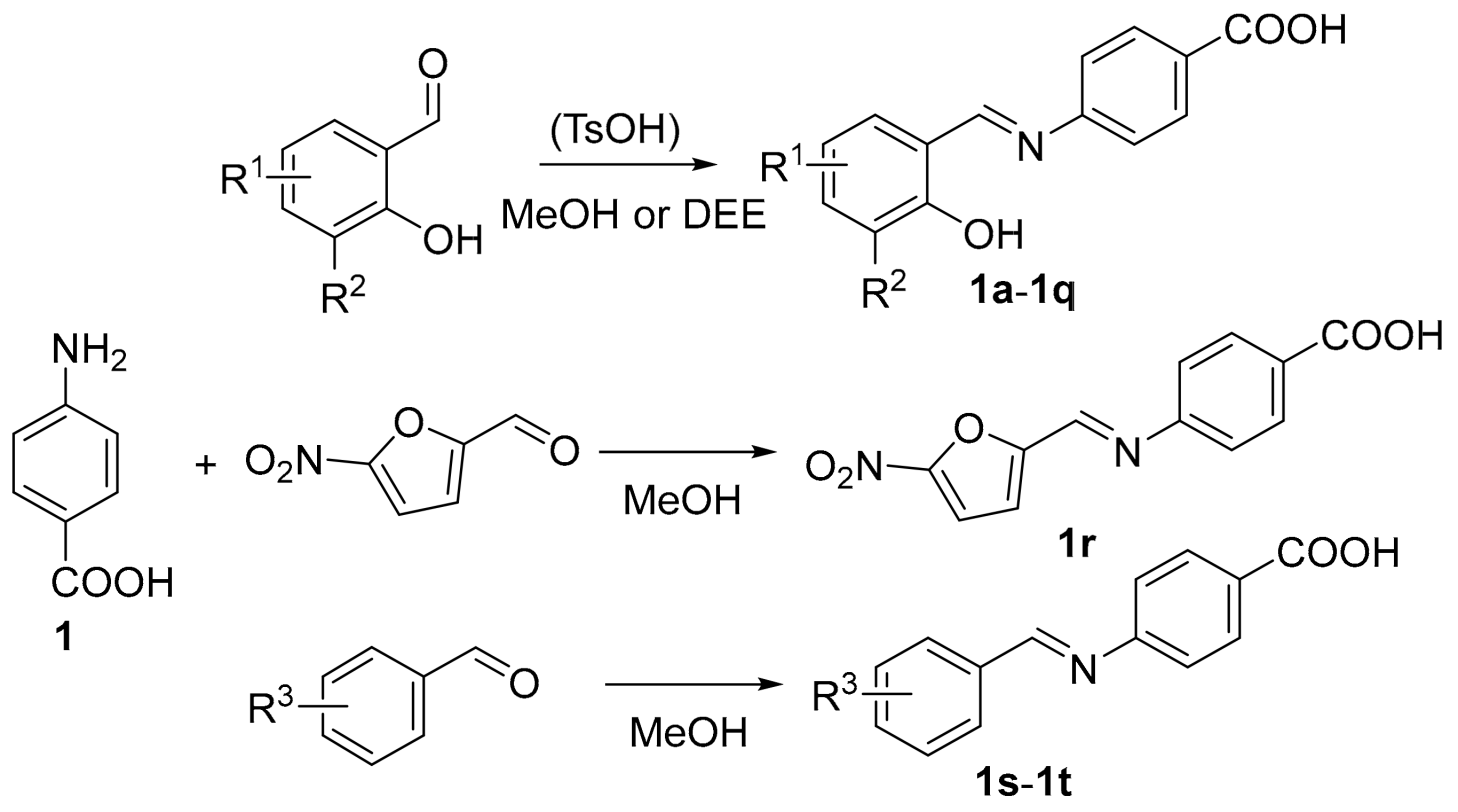
Synthesis of PABA-based imines **1a**–**1t** (R^1^ = H, 5-F, 4-Cl, 5-Cl, 6-Cl, 5-Br, 5-I, 5-NO_2_, 5-CH_3_, 5-OCH_3_, 5-OH, 5-*t*-Bu; R^2^ = H, Cl, Br, I; R^3^ = 2-OCH_3_, 4-OH; TsOH = toluene-4-sulfonic acid; DEE = diethyl ether).

**Scheme 2:**
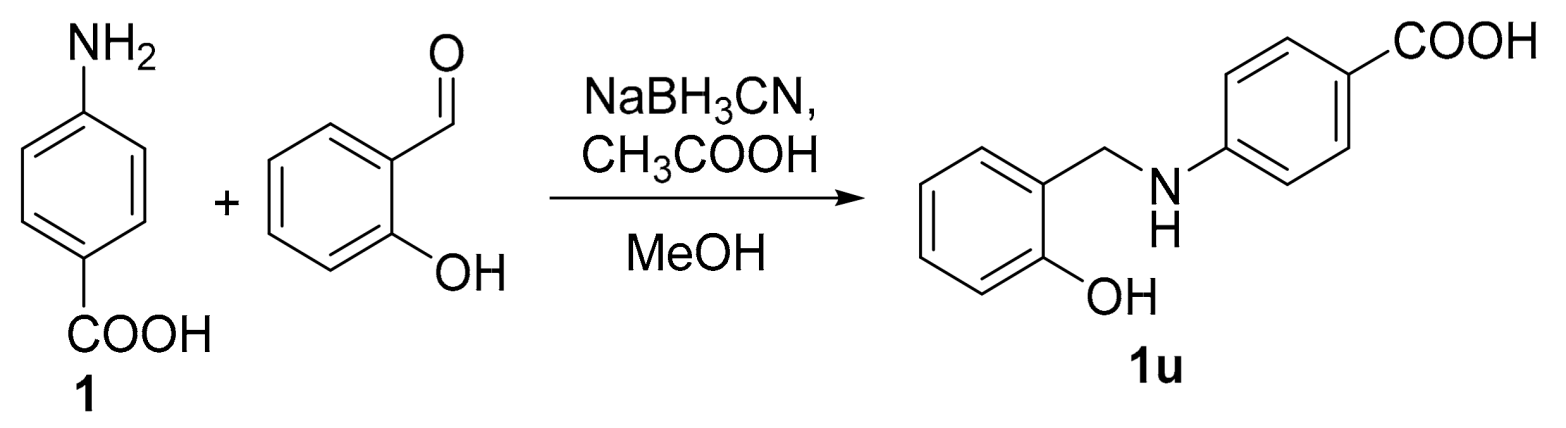
Synthesis of the derivative **1u** via reductive amination.

We synthesized twenty PABA-derived Schiff bases **1a**–**1t** and one “reduced” compound **1u** (i.e., **1a** with reduced imine double bond). The synthesis of Schiff bases (Scheme 1) involved a condensation of the amino acid with aldehydes in MeOH under reflux (3 h) or DEE at room temperature (72 h). Exceptionally, an acidic organic catalyst (0.1 equivalent of toluene-4-sulfonic acid monohydrate) was used to promote the reaction (imines **1f** and **1t**). The yields were from good to excellent (80–96%). Seventeen Schiff bases were prepared from substituted salicylaldehydes (**1a**–**1q**), one (**1r**) from 5-nitrofuran-2-carbaldehyde (5-nitrofurfural) and three compounds are analogues of **1a**: *O*-methyl derivative **1s**, 4-hydroxy isomer **1t** and molecule **1u** without double bond. Aforementioned 4-[(2-hydroxybenzyl)amino]benzoic acid **1u** was obtained by reductive amination from PABA and salicylaldehyde using sodium cyanoborohydride and glacial acetic acid in MeOH (yield 74%; Scheme 2). The structures of the aimed compounds are summarized in Table 1. 

New compounds were characterized by melting points, NMR and IR spectroscopy. Their purity was checked by the TLC and elemental analysis additionally. The identity of the known compounds was established using NMR and IR spectroscopy by the comparison with previously reported data. Their purity was checked in an identical way as for novel derivatives. 

### 3.2. Antibacterial Activity 

PABA derivatives **1a**–**1u** were evaluated for their antibacterial action using four Gram-positive strains of *Staphylococcus aureus* ATCC 29213, CCM 4223, methicillin-resistant *Staphylococcus aureus* ATCC 43300, CCM 4750 (MRSA), *Staphylococcus epidermidis* H 6966/08, *Enterococcus faecalis* ATCC 29212, CCM 4224 and four Gram-negative strains, *Escherichia coli* ATCC 25922, CCM 3954, *Klebsiella pneumoniae* D 11750/08, extended spectrum β-lactamase positive *K. pneumoniae* J 14368/08, and *Pseudomonas aeruginosa* ATCC 27853, CCM 3955 (Table 1). Bacitracin, neomycin and PABA itself were used for comparison of MIC, ciprofloxacin and gentamicin as internal quality standards (their MIC data are not shown).

Free PABA lacked any antibacterial properties (MIC > 500 µM). Generally, Gram-positive strains were more susceptible (MIC from 15.62 µM) than Gram-negative species (≥ 62.5 µM and majority of the compounds **1** share no activity). Illustratively, *P. aeruginosa* was inhibited only by two imines at 500 µM (**1i** and **1r**). On the other hand, *S. epidermidis* was found being the most susceptible strain (≥ 15.62 µM) followed by both drug-susceptible and methicillin-resistant *S. aureus*. Importantly, the growth inhibition of *S. aureus* is independent of the presence of methicillin-resistance, thus indicating no cross-resistance with this drug. The MIC values obtained for the most active imines are identical to those obtained for bacitracin, an antibiotic used for the treatment of topical infections caused by Gram-positive bacteria. Due to a lack of a significant activity of **1a**–**1u** against Gram-negative strains, none of the derivatives were superior to aminoglycoside drug neomycin.

The following structure-activity relationships (SAR) were identified:

Substituted salicylidene moiety is essential: derivatives of unsubstituted salicylaldehyde (**1a**) and its reduced analogue **1u**, 2-methoxybenzaldehyde (**1s**) and 4-hydroxybenzaldehyde (**1t**) avoided any antibacterial action.

1. The presence of 5-F (**1b**), 5-*t*-Bu (**1j**) and chlorine at the positions 4 and 6 of the salicylic ring (**1k**, **1l**) did not improve activity of **1a**.
2. Nitro (**1f**) and hydroxy group (**1i**) with opposite electron properties led to similar results—inhibition of multiple Gram-positive strains including MRSA, but at high concentrations (≥ 250 µM).
3. The 5-substitution by a single halogen (Cl **1c**, Br **1d**, I **1e**) improves the inhibition of *S. epidermidis*; in the case of **1e** MRSA, too (MIC from 62.5 µM).
4. Electron-donation groups (CH_3_ **1g**, OCH_3_ **1h**) together with 3-Cl (**1m**) produced inhibition of only *S. epidermidis*, but with MIC from 250 µM.
5. 5-Nitrofurylidene PABA derivative **1r** inhibited all three staphylococcal strains (MIC 62.5–125 µM).
6. The Schiff bases derived from 3,5-dihalogenosalicylaldehydes (**1n**–**1q**) represent the most successful modification of PABA with MIC values of ≥ 15.62 µM, especially if the particular halogens are different (**1o**, **1p**). These latter compounds containing one iodine atom also demonstrated activity against three Gram-negative strains (*E. coli*, *K. pneumoniae*).

In summary, the presence of at least one heavier halogen is essential, favouring derivatives of 5-chloro-3-iodo/bromo-salicylaldehydes. These substituents provide both wide-spectrum antibacterial activity and comparatively low MIC values. Interestingly, similar results were obtained for sulfadiazine Schiff bases previously [20]. MIC of 5-nitrofurfural-derived Schiff base **1r** (62.5–125 µM, i.e., 16.25–32.51 µg/mL) is fully comparable to breakpoint of its clinically used analogue nitrofurantoin for staphylococci (32–64 µg/mL) according to EUCAST [33].

Although PABA is an essential molecule for folate biosynthesis, it was reported as an agent inhibiting *E. coli*, *Listeria monocytogenes* and *Salmonella enteritidis* [6], but at supraphysiological concentrations. Although the strains used by Richards et al. are distinct from our study, their reported MIC values of PABA were substantially higher (9–24 mM) [6]. Giving together with the ineffectiveness of PABA in our assay, it is obvious that the antibacterial action depends on the presence of imine bond resulting from the reaction with substituted salicylaldehydes or 5-nitrofurfural, i.e., the bioactivity is established by this simple chemical modification of the vitamin unequivocally. The substitution of NH_2_ group by carbonyl group could interfere with the vitamin function of PABA and thus folate biosynthesis. Alternatively, PABA may be considered as a carrier for intrinsic bioactive salicylic and nitrofuran scaffolds. From this point of view, PABA that has demonstrated selective bacterial uptake (*S. aureus*, *E. coli*, *M. tuberculosis*) [37] should help to accumulate Schiff bases and thus salicylic and nitrofuran molecules within bacterial cells where they can disrupt cellular functions leading to growth inhibition or cellular death. 

### 3.3. Antimycobacterial Activity 

Schiff bases **1a**–**1t** and phenol **1u** were screened in vitro against *Mtb.* 331/88 (i.e., H_37_Rv) and three non-tuberculous mycobacterial strains: *M. avium* 330/88, *M. kansasii* 235/80 and *M. kansasii* 6509/96 (a clinical isolate). A first-line antitubercular drug INH and parent PABA were used as reference drugs for comparison (Table 2).

In general, although none of the compounds was inactive completely, antimycobacterial activity of the derivatives **1a**–**1u** is comparatively weak with MIC from 62.5 µM. 5-nitrofurfural derivative **1r** showed the lowest MIC (62.5–250 µM); only this derivative outstripped INH in the case of *M. avium* and *M. kansasii* 235/80 (i.e., INH-resistant strains). This finding was expected regarding previous studies, e.g., [38]. The Schiff bases derived from unsubstituted salicylaldehyde (**1a**), its 4-OH isomer (**1t**), 2-methoxybenzaldehyde (**1s**) and reduced compound **1u** share only negligible antimycobacterial activity corresponding to the parent PABA. The substitution of salicylaldehyde (compounds **1b**–**1q**; especially *tert*-butyl **1j** and halogenated imines **1i**, **1k**, **1l**, **1p** and **1q**) improved growth inhibition of mycobacteria, but only moderately.

### 3.4. Antifungal Activity 

PABA derivatives **1a**–**1u** were investigated also against eight fungal strains. The panel of human pathogens covers five yeast strains (*Candida albicans* ATCC 44859, *Candida tropicalis* 156, *Candida krusei* E28, *Candida glabrata* 20/I, *Trichosporon asahii* 1188) and three moulds (*Aspergillus fumigatus* 231, *Lichtheimia corymbifera* 272, and *Trichophyton interdigitale* 445). Azole drug fluconazole and parent PABA were used for comparison of MIC (Table 3).

PABA itself showed no antifungal properties (MIC > 500 µM). In general, *A. fumigatus* and *L. corymbifera* were found being the most resistant species (MIC from 125 µM). On the other hand, yeasts and *T. interdigitale* were inhibited from the concentration of 7.81 µM. Interestingly, the inhibition of these strains by particular derivatives were virtually identical. The PABA Schiff bases were superior to fluconazole for seven strains: *C. tropicalis* (**1c**–**1e**, **1h**, **1k**, **1m**–**1r**), *C. krusei* (inherent FLU-resistant; (**1e**, **1o**–**1r**; other derivatives were comparable—**1c**, **1d**, **1k**, **1m**, and **1n**), *C. glabrata* (**1d**, **1e**, **1m**, **1o**–**1q**), *T. asahii* (**1d**, **1e**, **1m**–**1q**; others are comparable: **1f**, **1h**, **1r**), *A. fumigatus* (**1d**, **1e**, **1m**–**1q**), *L. corymbifera* (**1f**, **1m**–**1q**) and *T. interdigitale* (**1p**, **1q**; additional derivatives were superior or comparable after 120 h of treatment).

These SAR were found:

1. Substituted salicylidene or 5-nitrofurfurylidene moiety is indispensable: unsubstituted salicylaldehyde (**1a**), its reduced analogue **1u**, 2-methoxybenzaldehyde (**1s**) and 4-hydroxybenzaldehyde (**1t**) share no antimycotic potency.
2. 5-F (**1b**) and 5-alkyls (**1g**, **1j**) are not translated into any antifungal action.
3. The presence of NO_2_ (**1f**), OH (**1i**), OCH_3_ (**1h**) and 4-Cl (**1l**) substituents provides active compounds but with higher MIC values (≥ 250 µM).
4. Focusing on isomeric 6-Cl (**1k**), 5-Cl (**1c**), 4-Cl (**1l**) and 3-Cl (**1m**) derivatives, the increase of the activity is as follows: 4-Cl (MIC ≥ 250 µM) < 5-Cl ≤ 6-Cl < 3-Cl (MIC ≥ 31.25 µM). Interestingly, the introduction of second chlorine atom (3,5-Cl_2_) did not improve the activity (**1n**; MIC ≥ 125 µM).
5. The activity of single halogen-substituted derivatives (5-F **1b**, 5-Cl **1c**, 5-Br **1d**, 5-I **1e**) is increasing with the atomic mass/lipophilicity/atomic radius of the halogens (5-F > 500 µM; 5-I ≥ 31.25 µM).
6. 3,5-Dihalogenosalicylidene imines (1n–1q) were identified as the most active derivatives providing both low MIC (≥ 7.81 µM) and broad-spectrum of the activity. The presence of at least one iodine atom results in the highest antifungal properties (**1o** and **1p**).

Obviously, SAR are predominantly analogous to antibacterial action. Recently, Laborda et al. [39] have discovered PABA as antifungal substance of *Lysobacter antibioticus* supressing phytopathogenic fungi, even though at high concentrations again (IC_50_ of 1–3 mM), i.e., being up to three orders of magnitude less potent then our compounds. The maximal concentration of PABA investigated in our study was lower than reported as effective [39]. To the best of our knowledge, no activity of PABA-based imines working against human pathogenic fungi has been reported and this property is presented herein for the first time. Again, a simple structural change of this vitamin-like molecule constitutes new bioactivity. 

Notably, Meir and Osherov [40] presented and highlighted vitamin biosynthesis as promising antifungal targets including folic acid biosynthesis utilizing PABA. The deficient synthesis of PABA and withdrawal from food hampered the virulence of *Aspergillus* spp. completely. Moreover, PABA inhibitors will likely be active against ongoing infections.

### 3.5. Cytotoxicity

The cytotoxicity of the PABA derivatives **1** were screened using the standard hepatic cancer cell line HepG2 (Table 4) from two reasons. First, salicylidene-based compounds have been proposed as anticancer agents supressing also HepG2 cells [41]. On the other hand, they can serve as an in vitro model for the hepatotoxicity as the surrogate of normal liver cells during preclinical drug discovery [42,43].

The CellTiter 96 assay used is based on the reduction of tetrazolium dye in living cells to formazan, which is then determined colorimetrically. This reduction is related to availability of NADH or NADPH. The decline in levels of these metabolically important compounds in the cell causes that the production of formazan is reduced. For quantitative comparison of cytotoxicity, the parameter IC_50_ was used. IC_50_ is defined as the concentration that reduces the viability of the cells to 50% of the maximal viability.

We were able to determine the exact IC_50_ of the majority of the substances. However, IC_50_ values of parent PABA **1**, **1a**, **1t** and **1u** were higher than tested range of concentrations. The measurement was not possible due to the precipitation of the compound in the medium. The IC_50_ of **1i** could not be determined with the used method due to strong interaction with the testing reagent.

The effective cytotoxic concentrations among the compounds differ in the extent of several orders of magnitude. We could identify both several relatively non-toxic compounds for HepG2 cells (IC_50_ > 500 µM: **1a**, **1g**, **1h**, **1s**, **1t**, **1u** and PABA **1**), other compounds exhibited moderate toxicity. 5-nitrofurylidene-based imine was identified as the most toxic compound (IC_50_ = 15.0 µM), followed by polyhalogenated derivatives and 5-*tert*-butyl compound **1j**. Interestingly, there are not identical SAR as for antimicrobial action: heavier/more lipophilic halogens do not result in uniformly enhanced toxicity and, regarding particular chlorinated isomers, 5-isomer is approximately twice more toxic than other. The IC_50_ value of the most cytotoxic compound (nitrofuran derivative **1r**) was fully comparable to those obtained for anticancer drugs tamoxifen (19.6 µM) and cisplatin (21.3 µM), other the most active compounds exhibited IC_50_ in the same order of magnitude (**1j**, **1n**–**1p**).

In addition to PABA “non-imine” cytotoxic derivatives [1,5,12,44], Farooq et al. [18] discovered *N*-alkylisatin-3-iminobenzoic acids, i.e., Schiff bases of substituted isatin and PABA, as potential cytotoxic/antileukemic agents with IC_50_ for human normal as well as cancer cell lines comparable to our the most active molecules. Thus, the formation of Schiff bases could convert non-toxic PABA to moderate cytotoxic agents, depending on the carbonyl compound used. Seemingly negligible changes to the structure may result in substantially higher toxicity for malignant cells (illustratively, switch of a lipophilic and electron-donating substituent methyl **1g** to *tert*-butyl **1j** increases toxicity almost twelve times). Since PABA as the parent compound itself is non-toxic and safe for human administration [8,9], the cytotoxicity should be a consequence of the properties of the attached aldehyde and/or imine bond.

## 4. Conclusions

In this study, we synthesized twenty-one hybrid compounds of folate precursor 4-aminobenzoic acid, an essential vitamin for many human pathogens, and bioactive aldehydes, predominantly substituted salicylaldehydes and 5-nitrofurfural. These scaffolds were fused through imine bond to form Schiff bases. Advantageously, the aimed compounds are easily synthetically available. 

The modification of PABA in this way led to constitution of various bioactivities that were not exhibited by the parent drug in our assays. SAR revealed following essential structural features: (1) presence of imine bond; (2) (5-nitrofuran-2-yl)methylene or 2-hydroxybenzylidene (salicylidene) scaffold; (3) substitution of salicylic moiety, especially by one or more heavier halogen(s). Many of the new compounds share antibacterial action especially against Gram-positive strains including MRSA, mild inhibition of mycobacteria and broad-spectrum antifungal properties. The most potent agents against Gram-positive strains were halogenated, especially 3,5-dihalogenated derivatives with MIC values from 15.62 µM. The 5-nitrofurfurylidene scaffold was proven to be beneficial for the inhibition of mycobacteria (MIC of ≥ 62.5 µM). Moreover, the simple chemical derivatization of non-toxic parent amino acid can result in compounds with cytotoxic action for cancer cells, but many derivatives remained non-toxic. The presence of heavier halogen(s), especially iodine, highly lipophilic *tert*-butyl and nitro group (IC_50_ from 15.0 µM) enhanced the cytotoxicity of the Schiff bases for HepG2 line. In sum, structure-activity relationships highlighted the presence of dihalogenated salicylic and 5-nitrofurane moieties. Regarding the carbonyl compound used for the modification of the PABA structure, it is possible to tune up the particular activities to identify even more promising derivatives for further development.

## Figures and Tables

**Table 1:** Antibacterial activity of PABA derivatives **1.**

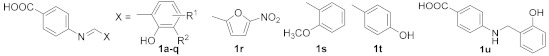																		
Code	R^1^	R^2^	MIC [µM]															
			SA		MRSA		SE		EF		EC		KP		KP-E		PA	
			24 h	48 h	24 h	48 h	24 h	48 h	24 h	48 h	24 h	48 h	24 h	48 h	24 h	48 h	24 h	48 h
**1a**	H	H	>500	>500	>500	>500	>500	>500	>500	>500	>500	>500	>500	>500	>500	>500	>500	>500
**1b**	5-F	H	>500	>500	>500	>500	>500	>500	>500	>500	>500	>500	>500	>500	>500	>500	>500	>500
**1c**	5-Cl	H	>500	>500	>500	>500	**62.5**	**62.5**	>500	>500	>500	>500	>500	>500	>500	>500	>500	>500
**1d**	5-Br	H	>250	>250	>250	>250	**62.5**	**62.5**	>250	>250	>250	>250	>250	>250	>250	>250	>250	>250
**1e**	5-I	H	>250	>250	250	250	**62.5**	**62.5**	>250	>250	>250	>250	>250	>250	>250	>250	>250	>250
**1f**	5-NO_2_	H	500	500	500	500	250	250	500	500	>500	>500	>500	>500	>500	>500	>500	>500
**1g**	5-CH_3_	H	>500	>500	>500	>500	250	250	>500	>500	>500	>500	>500	>500	>500	>500	>500	>500
**1h**	5-OCH_3_	H	>500	>500	>500	>500	500	500	>500	>500	>500	>500	>500	>500	>500	>500	>500	>500
**1i**	5-OH	H	500	500	250	250	250	250	500	500	>500	>500	500	500	500	500	**500**	**500**
**1j**	5-*tert*-Bu	H	>500	>500	>500	>500	>500	>500	>500	>500	>500	>500	>500	>500	>500	>500	>500	>500
**1k**	6-Cl	H	>500	>500	>500	>500	>500	>500	>500	>500	>500	>500	>500	>500	>500	>500	>500	>500
**1l**	4-Cl	H	>500	>500	>500	>500	>500	>500	>500	>500	>500	>500	>500	>500	>500	>500	>500	>500
**1m**	H	Cl	>500	>500	>500	>500	500	500	>500	>500	>500	>500	>500	>500	>500	>500	>500	>500
**1n**	5-Cl	Cl	125	125	250	250	250	250	>500	>500	>500	>500	>500	>500	>500	>500	>500	>500
**1o**	5-Cl	Br	**15.62**	**15.62**	**31.25**	**31.25**	**15.62**	**31.25**	**62.5**	**62.5**	500	500	500	500	500	500	>500	>500
**1p**	5-Cl	I	**15.62**	**15.62**	**15.62**	**15.62**	**31.25**	**31.25**	**62.5**	**62.5**	**62.5**	**62.5**	**125**	**125**	**125**	**125**	>500	>500
**1q**	5-I	I	62.5	62.5	62.5	62.5	62.5	62.5	500	500	500	500	500	500	500	500	>500	>500
**1r**	-	-	62.5	62.5	62.5	62.5	125	125	>500	>500	>500	>500	500	500	500	500	**500**	**500**
**1s**	-	-	>500	>500	>500	>500	>500	>500	>500	>500	>500	>500	>500	>500	>500	>500	>500	>500
**1t**	-	-	>500	>500	>500	>500	>500	>500	>500	>500	>500	>500	>500	>500	>500	>500	>500	>500
**1u**	-	-	>500	>500	>500	>500	>500	>500	>500	>500	>500	>500	>500	>500	>500	>500	>500	>500
**PABA 1**	-	-	>500	>500	>500	>500	>500	>500	>500	>500	>500	>500	>500	>500	>500	>500	>500	>500
**BAC**	-	-	7.81	15.62	15.62	15.62	15.62	31.25	15.62	62.5	>500	>500	>500	>500	>500	>500	>500	>500
**NEO**	-	-	ND	ND	ND	ND	ND	ND	ND	ND	3.9	3.9	1.95	1.95	3.9	3.9	7.81	15.62

BAC = bacitracin; NEO = neomycin; PABA = 4-aminobenzoic acid. SA: *Staphylococcus aureus* CCM 4516/08; MRSA: methicillin-resistant *Staphylococcus aureus* H 5996/08; SE: *Staphylococcus epidermidis* H 6966/08; EF: *Enterococcus faecalis* J 14365/08. EC: *Escherichia coli* CCM 4517; KP: *Klebsiella pneumoniae* D 11750/08; KP-E: ESBL-positive *Klebsiella pneumoniae* J 14368/08; PA: *Pseudomonas aeruginosa* CCM 1961. ND = not determined. One or two of the best MIC value(s) for each strain are shown in bold.

**Table 2:** Antimycobacterial activity of PABA derivatives **1.**

Code	R	R^2^	MIC [µM]									
			*Mtb.* 331/88		*M. avium* 330/88		*M. kansasii* 235/80			*M. kansasii* 6509/96		
			14 d	21 d	14 d	21 d	7 d	14 d	21 d	7 d	14 d	21 d
**1a**	H	H	>1000	>1000	>1000	>1000	1000	>1000	>1000	1000	1000	1000
**1b**	5-F	H	500	500	>1000	>1000	250	500	500	500	500	500
**1c**	5-Cl	H	250	500	1000	>1000	250	250	500	500	500	1000
**1d**	5-Br	H	250	500	500	500	250	250	500	500	500	500
**1e**	5-I	H	250	250	500	500	250	250	250	**250**	**250**	500
**1f**	5-NO_2_	H	250	500	1000	>1000	500	500	500	500	500	500
**1g**	5-CH_3_	H	500	500	>1000	>1000	500	500	500	500	500	1000
**1h**	5-OCH_3_	H	250	500	1000	1000	500	500	500	500	500	500
**1i**	5-OH	H	500	1000	1000	>1000	500	500	1000	500	1000	1000
**1j**	5-*tert*-Bu	H	250	250	1000	>1000	**125**	**125**	250	**250**	**250**	500
**1k**	6-Cl	H	250	500	500	1000	250	500	500	**250**	**250**	500
**1l**	4-Cl	H	250	500	1000	1000	250	500	500	**250**	**250**	500
**1m**	H	Cl	500	500	1000	1000	500	500	500	**250**	500	500
**1n**	5-Cl	Cl	500	500	1000	1000	500	500	500	**250**	500	500
**1o**	5-Cl	Br	250	500	500	500	250	500	500	500	500	500
**1p**	5-Cl	I	250	250	500	1000	250	500	500	**250**	**250**	500
**1q**	5-I	I	250	250	500	500	250	500	500	**250**	**250**	**250**
**1r**	-	-	**62.5**	**125**	**250**	500	**62.5**	**125**	250	**125**	**125**	**250**
**1s**	-	-	1000	1000	>1000	>1000	1000	1000	>1000	1000	1000	1000
**1t**	-	-	1000	1000	1000	>1000	1000	1000	>1000	1000	1000	1000
**1u**	-	-	1000	1000	>1000	>1000	1000	1000	1000	1000	1000	1000
**PABA 1**	-	-	>1000	>1000	>1000	>1000	1000	>1000	>1000	1000	1000	1000
**INH**	-	-	0.5	1	>250	>250	>250	>250	>250	8	8	8

INH = isoniazid; PABA = 4-aminobenzoic acid; *Mtb.* = *Mycobacterium tuberculosis*. One or two of the lowest MIC value(s) for each strain are shown in bold.

**Table 3:** Antifungal activity of PABA derivatives **1.**

Code	R^1^	R^2^	MIC [µM]															
			CA		CT		CK		CG		TA		AF		LC		TI	
			24 h	48 h	24 h	48 h	24 h	48 h	24 h	48 h	24 h	48 h	24 h	48 h	24 h	48 h	72 h	120 h
**1a**	H	H	>500	>500	>500	>500	>500	>500	>500	>500	>500	>500	>500	>500	>500	>500	>500	>500
**1b**	5-F	H	>500	>500	>500	>500	>500	>500	>500	>500	>500	>500	>500	>500	>500	>500	>500	>500
**1c**	5-Cl	H	>125	>125	125	125	125	125	125	125	>125	>125	>125	>125	>125	>125	125	125
**1d**	5-Br	H	62.5	62.5	125	125	125	125	62.5	62.5	125	125	250	250	>250	>250	62.5	62.5
**1e**	5-I	H	62.5	62.5	125	125	62.5	62.5	31.25	31.25	62.5	62.5	**125**	**125**	>125	>125	**31.25**	**31.25**
**1f**	5-NO_2_	H	500	500	>500	>500	>500	>500	>500	>500	500	500	>500	>500	500	500	500	500
**1g**	5-CH_3_	H	>125	>125	>125	>125	>125	>125	>125	>125	>125	>125	>125	>125	>125	>125	>125	>125
**1h**	5-OCH_3_	H	250	250	500	500	500	500	250	250	500	500	>500	>500	>500	>500	>500	>500
**1i**	5-OH	H	500	500	>500	>500	>500	>500	250	250	>500	>500	>500	>500	>500	>500	>500	>500
**1j**	5-*tert*-Bu	H	>500	>500	>500	>500	>500	>500	>500	>500	>500	>500	>500	>500	>500	>500	>500	>500
**1k**	6-Cl	H	125	125	62.5	125	125	250	125	125	125	>125	>125	>125	>125	>125	125	125
**1l**	4-Cl	H	250	>500	500	>500	500	>500	500	>500	500	>500	250	>500	>500	>500	500	>500
**1m**	H	Cl	**31.25**	**31.25**	62.5	62.5	250	250	62.5	62.5	125	125	250	250	500	500	125	125
**1n**	5-Cl	Cl	125	125	250	250	125	125	125	125	125	125	500	500	500	500	250	250
**1o**	5-Cl	Br	**31.25**	**31.25**	62.5	62.5	62.5	62.5	**15.62**	**15.62**	31.25	31.25	250	250	500	500	62.5	62.5
**1p**	5-Cl	I	**7.81**	**7.81**	**31.25**	**31.25**	**15.62**	**15.62**	**7.81**	**7.81**	**7.81**	**7.81**	**125**	**125**	**250**	**250**	**7.81**	**7.81**
**1q**	5-I	I	**7.81**	**7.81**	**15.62**	**15.62**	**31.25**	**31.25**	**7.81**	**7.81**	**15.62**	**15.62**	**125**	**125**	**250**	**250**	**7.81**	**7.81**
**1r**	-	-	250	250	250	250	62.5	62.5	250	250	500	500	>500	>500	>500	>500	250	250
**1s**	-	-	>500	>500	>500	>500	>500	>500	>500	>500	>500	>500	>500	>500	>500	>500	>500	>500
**1t**	-	-	>500	>500	>500	>500	>500	>500	>500	>500	>500	>500	>500	>500	>500	>500	>500	>500
**1u**	-	-	>500	>500	>500	>500	>500	>500	>500	>500	>500	>500	>500	>500	>500	>500	>500	>500
**PABA 1**	-	-	>500	>500	>500	>500	>500	>500	>500	>500	>500	>500	>500	>500	>500	>500	>500	>500
**FLU**	-	-	0.24	0.24	>500	>500	125	250	31.25	500	250	500	>500	>500	>500	>500	7.81	125

FLU = fluconazole; PABA = 4-aminobenzoic acid. CA: Candida albicans ATCC 44859; CT: Candida tropicalis; CK: Candida krusei E28; CG: Candida glabrata 20/I; TA: Trichosporon asahii 1188; AF: Aspergillus fumigatus 231; LC: Lichtheimia corymbifera 272; TI: Trichophyton interdigitale 445. One or two of the best MIC value(s) for each strain are shown in bold.

**Table 4:** Cytotoxicity of the compounds **1** for HepG2 cells.

Code	R^1^	R^2^	IC_50_ [µM]	Range of Concentrations Tested
**1a**	H	H	>1500 *	1–1500
**1b**	5-F	H	497.6	1–500
**1c**	5-Cl	H	142.9	1–500
**1d**	5-Br	H	178.7	1–500
**1e**	5-I	H	113.4	1–500
**1f**	5-NO_2_	H	243.8	1–500
**1g**	5-CH_3_	H	956.4	1–1500
**1h**	5-CH_3_O	H	530.4	1–1500
**1i**	5-OH	H	ND	1–1500
**1j**	5-*tert*-Bu	H	80.8	1–500
**1k**	6-Cl	H	279.6	1–500
**1l**	4-Cl	H	263.9	1–500
**1m**	H	Cl	247.4	1–500
**1n**	5-Cl	Cl	98.7	1–500
**1o**	5-Cl	Br	85.7	1–500
**1p**	5-Cl	I	53.4	1–500
**1q**	5-I	I	57.8	1–500
**1r**	-	-	15.0	1–500
**1s**	-	-	1090.0	1–1500
**1t**	-	-	>1500 *	1–1500
**1u**	-	-	>500 *	1–500
**Tamoxifen**	-	-	19.6	1–500
**Cisplatin**	-	-	21.3	1–500
**PABA 1**	-	-	>1500 *	1–1500

* measurement at higher concentration was unable due to the precipitation of the compound in the medium. PABA = 4-aminobenzoic acid; ND = not determined.
